# Prescription Patterns for Tigecycline in Severely Ill Patients for Non-FDA Approved Indications in a Developing Country: A Compromised Outcome

**DOI:** 10.3389/fmicb.2017.00497

**Published:** 2017-03-27

**Authors:** Rima A. Moghnieh, Dania I. Abdallah, Ismail A. Fawaz, Tarek Hamandi, Mohammad Kassem, Nabila El-Rajab, Tamima Jisr, Anas Mugharbil, Nabila Droubi, Samaa Al Tabah, Loubna Sinno, Fouad Ziade, Ziad Daoud, Ahmad Ibrahim

**Affiliations:** ^1^Division of Infectious Diseases, Department of Internal Medicine, Makassed General HospitalBeirut, Lebanon; ^2^Faculty of Medicine, Beirut Arab UniversityBeirut, Lebanon; ^3^Faculty of Medical Sciences, Lebanese UniversityBeirut, Lebanon; ^4^Pharmacy Department, Makassed General HospitalBeirut, Lebanon; ^5^Department of Infectious Diseases, University of BalamandAmioun, Lebanon; ^6^Department of Internal Medicine, Makassed General HospitalBeirut, Lebanon; ^7^Department of Laboratory Medicine, Makassed General HospitalBeirut, Lebanon; ^8^Division of Hematology-Oncology, Department of Internal Medicine, Makassed General HospitalBeirut, Lebanon; ^9^Faculty of Health Sciences, American University of BeirutBeirut, Lebanon; ^10^Makassed General HospitalBeirut, Lebanon; ^11^Faculty of Public Health, Lebanese UniversityBeirut, Lebanon; ^12^Clinical Microbiology, Faculty of Medicine and Medical Sciences, University of BalamandAmioun, Lebanon

**Keywords:** tigecycline, critically ill, off-label indications, *Acinetobacter baumannii*, antibiotic resistance, antibiotic stewardship

## Abstract

**Introduction:** With the rise in antibiotic resistance, tigecycline has been used frequently in off-label indications, based on its *in-vitro* activity against multidrug-resistant organisms. In this study, our aim was to assess its use in approved and unapproved indications.

**Materials and Methods:** This is a retrospective chart review evaluating a 2-year experience of tigecycline use for > 72 h in 153 adult patients inside and outside critical care unit from January 2012 to December 2013 in a Lebanese tertiary-care hospital.

**Results:** Tigecycline was mostly used in off-label indications (81%) and prescribed inside the critical care area, where the number of tigecycline cycles was 16/1,000 patient days. Clinical success was achieved in 43.4% of the patients. In the critically ill group, it was significantly higher in patients with a SOFA score <7 using multivariate analysis (Odds Ratio (OR) = 12.51 [4.29–36.51], *P* < 0.0001). Microbiological success was achieved in 43.3% of patients. Yet, the univariate and adjusted multivariate models failed to show a significant difference in this outcome between patients inside vs. outside critical care area, those with SOFA score <7 vs. ≥ 7, and in FDA-approved vs. off-label indications. Total mortality reached ~45%. It was significantly higher in critically ill patients with SOFA score ≥7 (OR = 5.17 [2.43–11.01], *P* < 0.0001) and in off-label indications (OR = 4.00 [1.30–12.31], *P* = 0.01) using an adjusted multivariate model. Gram-negative bacteria represented the majority of the clinical isolates (81%) and *Acinetobacter baumannii* predominated (28%). Carbapenem resistance was present in 85% of the recovered *Acinetobacter*, yet, more than two third of the carbapenem-resistant *Acinetobacter* species were still susceptible to tigecycline.

**Conclusion:** In our series, tigecycline has been mostly used in off-label indications, specifically in severely ill patients. The outcome of such infections was not inferior to that of FDA-approved indications, especially inside critical care area. The use of this last resort antibiotic in complicated clinical scenarios with baseline microbiological epidemiology predominated by extensively-drug resistant pathogens ought to be organized.

## Introduction

The emergence of antimicrobial resistance among gram-positive and gram-negative organisms has led to the famous “bad bugs, no drugs” situation encountered primarily with the “ESKAPE” pathogens (Boucher et al., 2009). The paucity of active agents against organisms such as extensive drug-resistant (XDR) *Acinetobacter baumannii* (XDR-AB), carbapenem-resistant *Enterobacteriaceae* (CRE), and carbapenem-resistant *Pseudomonas aeruginosa* has led to the use of old antibiotics that were previously abandoned because of their toxicity, such as polymyxins, or the premature use of new antimicrobials in indications where they have not yet been proved to be active (Bassetti and Righi, 2015).

Tigecycline, the first in-class glycylcycline, was first approved by the Food and Drug Administration (FDA) and then by the European Medicines Agency in 2005 and 2006, respectively, for the treatment of complicated intra-abdominal infections (cIAIs; Babinchak et al., 2005) and complicated skin and soft-tissue infections (cSSTIs) in adults (Ellis-Grosse et al., 2005). In 2009, the FDA added community-acquired bacterial pneumonia (CAP) in adults to the list of approved indications (Tanaseanu et al., 2008). Tigecycline has demonstrated a promising *in vitro* activity against extended spectrum beta-lactamase (ESBL)-producing *Enterobacteriaceae*, CRE, and XDR-AB (Mendes et al., 2010; Balode et al., 2013; Sader et al., 2015). Because of the absence of other treatment options, it has been administered off-label for treating infections including hospital-acquired pneumonia (HAP), ventilator-associated pneumonia (VAP), urinary tract infections (UTI), sepsis, bacteremia, and febrile neutropenia (De Rosa et al., 2015).

Tigecycline has been widely used in our healthcare institution for the management of hospital-acquired infections caused by multidrug-resistant bacteria, without a specified protocol. Whether it should be used as a last-resort treatment option for such indications remains a complex issue that necessitates ongoing assessment. We retrospectively reviewed the prescribing pattern of tigecycline in a real-life clinical situation. We correlated its use to the microbiological ecology in this healthcare setting with regard to the prevalent XDR gram-negative resistance.

## Materials and methods

### Study design and patients

This is a retrospective chart review conducted at Makassed General Hospital, a 200-bed university hospital located in Beirut, Lebanon, with a 13-bed critical care unit, between January 2012 and December 2013. It included 153 adult patients who received tigecycline for more than 72 h. The hospital's Institutional Review Board committee approved this study. All patients received the standard FDA-approved dosage of tigecycline: a loading dose of 100 mg, followed by 50 mg administered intravenously every 12 h.

We recorded baseline clinical and demographic characteristics, microbiological findings, indication for tigecycline therapy, concomitant use of other antibiotics, clinical and microbiological outcome, and mortality. FDA-approved indications were CAP, cSSTI, and cIAI. Off-label indications were HAP, VAP, UTI, diabetic ulcers, sepsis, bacteremia, and febrile neutropenia. Severity of underlying disease was assessed with sequential organ failure assessment (SOFA) score for critically ill patients.

### Definitions

Infections were defined according to clinical diagnostic criteria established by the Center of Disease Control and Prevention and other international guidelines (Garner et al., 1988; Centers for Disease Control Prevention, 2012; Dellinger et al., 2013). Infections were classified as nosocomial acquired when onset occurred more than 48 h after hospital admission, and the infection was not present or incubating at that time (Garner et al., 1988). Infections occurring within 48 h of admission to the hospital were considered community-acquired, unless the patient had been transferred directly from another healthcare facility or discharged within the 30 days preceding hospital admission (Garner et al., 1988).

Empiric use of tigecycline was defined as its administration to a patient with signs and symptoms of infection without an identified source or a specific microbiological isolate (Bassetti et al., 2010). Targeted therapy was defined as tigecycline administration in the presence of an identified isolate (Bassetti et al., 2010).

As recommended by the World health Organization (WHO), tigecycline consumption was reported in Defined Daily Doses (DDD)/100 Bed Days (BD), a standardized figure that provides a degree of comparison among in-patients in different hospitals (World Health Organization, 2003). DDD corresponds to the assumed average daily dose of a specific drug for its main indication in adults (World Health Organization, 2003). Number of BD = Number of beds × occupancy index × Number of days (during that period). A bed-day corresponds to one occupied hospital bed during 1 day (World Health Organization, 2003).

### Microbiological studies and breakpoints of resistance

Bacterial identification was performed according to standard microbiological procedures. Antibiotic susceptibility was performed using the disc diffusion method as recommended by the Clinical and Laboratory Standards Institute (CLSI) for all organisms (CLSI, 2016). Quality-control strains used in the testing of *Acinetobacter* species and *P. aeruginosa*, and the *Enterobacteriaceae* were *E. coli* American Type Culture Collection (ATCC) 25922 and *P. aeruginosa* ATCC 27853. Acinetobacter species susceptibility was tested against all antimicrobials enlisted in CLSI guidelines (CLSI, 2016) except for ampicillin-sulbactam since it is non-available in Lebanon. Extensive drug resistance in *A. baumannii* was defined as non-susceptibility to at least one agent in all but susceptible to only one or two categories (Magiorakos et al., 2012). In our center, isolates were considered XDR if they were susceptible to tigecycline and colistin only and resistant to the following categories: aminoglycosides, antipseudomonal carbapenems, antipseudomonal FQs, extended-spectrum cephalosporins, antipseudomonal penicillins plus beta-lactamase inhibitors, monobactams and trimethoprim/sulfamethoxazole (TMP/SMX).

A 15-μg disc of tigecycline (Oxoid Ltd, Cambridge, UK) was used to determine susceptibility, and the breakpoints suggested by the FDA for *Enterobacteriaceae* (susceptible ≥19 mm; intermediate 15–18 mm, and resistant ≤ 14 mm), and gram-positive microorganisms (susceptible ≥19 mm) were used (Tygacil package insert, June 2005, Wyeth Pharmaceuticals, Inc., Philadelphia, PA). Tigecycline clinical breakpoints using the disc-diffusion method against *A. baumanii* have not been established by the CLSI (2016) or by the European Committee on Antimicrobial Susceptibility Testing (The European Committee on Antimicrobial Susceptibility Testing, 2016). Therefore, we applied the clinical breakpoints suggested by Jones et al. (2007) (susceptible ≥16 mm, intermediate 13–15 mm, and resistant ≤ 12 mm). Our medical laboratory department does not routinely perform broth microdilution method and a reference laboratory is not available in our country. However, during the study period, 11 randomly selected strains of *A. baumanii* were sent to another clinical microbiology laboratory (Faculty of Medicine and Medical Sciences, University of Balamand, Amioun, Lebanon) at a rate of one strain per 45 days when available, where broth microdilution method was performed for evaluating colistin and tigecycline susceptibility. We found 100% correlation between results of disc diffusion and broth microdilution (Supplementary File 1).

### Clinical outcome

Clinical success was defined as a positive response (partial or complete improvement of signs/symptoms of infection) and the lack of need to use a new antibiotic for the initial infection for 72 h after the discontinuation of tigecycline (Kuo et al., 2011; Montravers et al., 2014). Persistence or deterioration of the initial infection symptoms/signs requiring a change of antibiotic therapy, an infection-related death occurring later than 48 h after the start of tigecycline, and/or premature treatment discontinuation because of a tigecycline-related adverse event were considered clinical failures (Montravers et al., 2014; Kuo et al., 2011). Cases of insufficient data, death not directly related to the initial infection or occurring within the first 48 h of treatment, or the addition of an antibacterial agent for another infection (Montravers et al., 2014) were considered to be an undetermined clinical response.

### Microbiological outcome

Microbiological success was defined as the eradication/sterile culture results during or after the course of antibiotic therapy (Montravers et al., 2014). The persistent identification of the same organism for 72 h after initiation of antibiotic treatment was considered a microbiological failure (Montravers et al., 2014). Superinfection was defined as a new isolate obtained during the treatment of the original infection with development of symptoms and signs of clinical infection (Kuo et al., 2011). A specimen that was not available for estimation of eradication was considered an unavailable response (Montravers et al., 2014). Because our study was retrospective, microbiological success rates were calculated when follow-up cultures were available.

### Mortality

Seven days post-treatment mortality, a surrogate marker for treatment failure, was defined as death occurring within 48 h up to 7 days after the end of tigecycline therapy. In-hospital mortality, a surrogate marker of the general patient condition, was defined as the death occurring between 7 and 30 days after finishing tigecycline therapy. Total mortality was the sum of both 7 days post-treatment mortality and in-hospital mortality.

### Statistical analysis

The Statistical Package for Social Sciences program (IBM SPSS Statistics for Windows, Version 23.0, IBM Corp., Armonk, NY, USA) was used for data entry, management, and analyses. Descriptive analysis was carried out for categorical variables by number and percent. We assessed through univariate analysis the significance of baseline characteristics, comorbidities, types of infections, treatment strategy, outcome and mortality in patients who received tigecycline inside vs. outside critical care, and those with SOFA score <7 vs. those with SOFA score ≥7 inside ICU. Univariate analysis for the groups was carried out using the Chi square test for categorical variables, and independent *t*-test for continuous ones. This was followed by multivariate logistic regression for outcome and mortality to assess further significance between the groups, while controlling for potentially confounding variables. Parameters with *P* < 0.05 at the univariate level and other clinically significant variables were included in the multivariate model. Results were presented as odds ratio (OR) and 95% confidence interval (CI). We also performed univariate and multivariate analyses for outcome and mortality in FDA-approved indications vs. off-label indications.

## Results

### Patients' clinical characteristics

In total, 153 patients received tigecycline therapy within our healthcare facility between January 2012 and December 2013. The clinical characteristics of the patients are reported in Table 1. Most of them were elderly, with an age above 65 years (53%). This population suffered from multiple comorbidities, including cardiovascular disease (59%), diabetes (30%), acute renal failure (23%), and hemodynamic insufficiency (14%). One third of our patients (30.7%) were critically ill, thus justifying the use of intravenous catecholamines (24%) and mechanical ventilation (23%).

**Table 1:** **Univariate analysis for baseline characteristics, comorbidities, types of infections, and treatment strategy in patients who received tigecycline inside and outside critical care, and those with SOFA score <7 and ≥7 inside critical care during the study period (*N* = 153 patients)**.

**Clinical characteristics and treatment strategy**	**Total (*N* = 153)**	**Critically ill (*n* = 47)**	**Non-critically ill (*n* = 106)**	***P***	**Critically ill with SOFA < 7 (*n* = 17)**	**Critically ill with SOFA ≥7 (*n* = 30)**	***P***
**AGE (YEARS)**							
<45	35 (22.9%)	12 (25.5%)	23 (21.7%)	0.09	7 (41.2%)	5 (16.7%)	0.02
>45 and ≤ 65	37 (24.2%)	6 (12.8%)	31 (29.2%)		4 (23.5%)	2 (6.7%)	
>65	81 (52.9%)	29 (61.7%)	52 (49.1%)		6 (35.3%)	23 (76.7%)	
**GENDER**							
Male	79 (51.6%)	23 (48.9%)	56 (52.8%)	0.66	6 (35.3%)	17 (56.7%)	0.16
Female	74 (48.4%)	24 (51.1%)	50 (47.2%)		11 (64.7%)	13 (43.3%)	
**COMORBIDITIES AND RISK FACTORS**							
Cardiovascular Disease	90 (58.8%)	36 (76.6%)	54 (50.9%)	0.003	10 (58.8%)	26 (86.7%)	0.03
Diabetes	46 (30.1%)	19 (40.4%)	27 (25.5%)	0.06	5 (29.4%)	14 (46.7%)	0.25
COPD	21 (13.7%)	10 (21.3%)	11 (10.4%)	0.07	4 (23.5%)	6 (20.0%)	0.78
ARF	35 (22.9%)	12 (25.5%)	23 (21.7%)	0.60	1 (5.9%)	11 (36.7%)	0.02
CKD	19 (12.4%)	7 (14.9%)	12 (11.3%)	0.54	1 (5.9%)	6 (20.0%)	0.19
CHD	6 (3.9%)	0 (0.0%)	6 (5.7%)	0.10	0 (0.0%)	0 (0.0%)	NA
Hemodynamic Failure	22 (14.4%)	7 (14.9%)	15 (14.2%)	0.90	1 (5.9%)	6 (20.0%)	0.19
Vasopressors given before or ≤ 24 h of tigecycline therapy	37 (24.2%)	18 (38.3%)	19 (17.9%)	0.01	2 (11.8%)	16 (53.3%)	0.005
Mechanical ventilation	35 (22.9%)	17 (36.2%)	18 (17.0%)	0.01	4 (23.5%)	13 (43.3%)	0.17
Neutropenia (<500 mm^3^)	30 (19.6%)	2 (4.3%)	28 (26.4%)	0.001	0 (0.0%)	2 (6.7%)	0.28
**MALIGNANCY**							
No cancer	103 (67.3%)	45 (95.7%)	58 (54.7%)	<0.0001	17 (100.0%)	28 (93.3%)	0.55
Leukemia	24 (15.7%)	1 (2.1%)	23 (21.7%)		0 (0.0%)	1 (3.3%)	
Lymphoma	11 (7.2%)	0 (0.0%)	11 (10.4%)		0 (0.0%)	0 (0.0%)	
Solid Tumor	15 (9.8%)	1 (2.1%)	14 (13.2%)		0 (0.0%)	1 (3.3%)	
**HSCT**							
No	139 (90.8%)	47 (100.0%)	92 (86.8%)		17 (100.0%)	30 (100.0%)	NA
Autologous	4 (2.6%)	0 (0.0%)	4 (3.8%)	0.03	0 (0.0%)	0 (0.0%)	
Allogeneic	10 (6.5%)	0 (0.0%)	10 (9.4%)		0 (0.0%)	0 (0.0%)	
**DURATION OF TIGECYCLINE THERAPY**							
72 h–10 days	98 (64.1%)	31 (66.0%)	67 (63.2%)	0.76	11 (64.7%)	20 (66.7%)	0.92
≥11–14 days	28 (18.3%)	7 (14.9%)	21 (19.8%)		3 (17.6%)	4 (13.3%)	
≥15 days	27 (17.6%)	9 (19.1%)	18 (17.0%)		3 (17.6%)	6 (20%)	
**INDICATIONS FOR TIGECYCLINE THERAPY**							
FDA approved Indications	29 (19.0%)	6 (12.8%)	23 (21.7%)	0.19	6 (35.3%)	0 (0.0%)	<0.0001
cSSTI	18 (11.8%)	2 (4.3%)	16 (15.1%)	0.055	2 (11.8%)	0 (0.0%)	0.055
cIAI	4 (2.6%)	0 (0.0%)	4 (3.8%)	0.18	0 (0.0%)	0 (0.0%)	NA
CAP	8 (5.2%)	4 (8.5%)	4 (3.8%)	0.22	4 (23.5%)	0 (0.0%)	0.005
Off-label Indications	124 (81.0%)	41 (87.2%)	83 (78.3%)	0.19	11 (64.7%)	30 (100.0%)	<0.0001
HAP	30 (19.6%)	11 (23.4%)	19 (17.9%)	0.43	2 (11.8%)	9 (30.0%)	0.16
VAP	39 (25.5%)	24 (51.1%)	15 (14.2%)	<0.0001	8 (47.1%)	16 (53.3%)	0.68
Bacteremia	16 (10.5%)	7 (14.9%)	9 (8.5%)	0.23	3 (17.6%)	4 (13.3%)	0.69
Sepsis	14 (9.2%)	2 (4.3%)	12 (11.3%)	0.16	0 (0.0%)	2 (6.7%)	0.28
Diabetic Ulcer	8 (5.2%)	2 (4.3%)	6 (5.7%)	0.72	1 (5.9%)	1 (3.3%)	0.68
UTI	6 (3.9%)	1 (2.1%)	5 (4.7%)	0.45	0 (0.0%)	1 (3.3%)	0.45
FN	24 (15.7%)	1 (2.1%)	23 (21.7%)	0.002	0 (0.0%)	1 (3.3%)	0.45
**TREATMENT STRATEGY**							
Empiric	67 (43.8%)	15 (31.9%)	52 (49.1%)	0.049	5 (29.4%)	10 (33.3%)	0.78
Targeted	86 (56.2%)	32 (68.1%)	54 (50.9%)		12 (70.6%)	20 (66.7%)	
Monotherapy	17 (11.1%)	4 (8.5%)	13 (12.3%)	0.50	1 (5.9%)	3 (10.0%)	0.63
Combination therapy	136 (88.9%)	43 (91.5%)	93 (87.7%)		16 (94.1%)	27 (90.0%)	
TGC+3GC	3 (2.0%)	0 (0.0%)	3 (2.8%)	0.24	0 (0.0%)	0 (0.0%)	NA
TGC+4GC	23 (15.0%)	5 (10.6%)	18 (17.0%)	0.31	3 (17.6%)	2 (6.7%)	0.24
TGC+TZP	38 (24.8%)	16 (34.0%)	22 (20.8%)	0.08	7 (41.2%)	9 (30.0%)	0.44
TGC+CAR	71 (46.4%)	27 (57.4%)	44 (41.5%)	0.07	8 (47.1%)	19 (63.3%)	0.28
TGC+CST	47 (30.7%)	16 (34.0%)	31 (29.2%)	0.55	6 (35.3%)	10 (33.3%)	0.89
TGC+AMG	29 (19.0%)	3 (6.4%)	26 (24.5%)	0.01	3 (17.6%)	0 (0.0%)	0.02
TGC+TZP+CST	12 (7.8%)	4 (8.5%)	8 (7.5%)	0.84	1 (5.9%)	3 (10.0%)	0.63
TGC+CAR+CST	26 (17.0%)	10 (21.3%)	16 (15.1%)	0.35	4 (23.5%)	6 (20.0%)	0.78
TGC+AMG+CST	11 (7.2%)	3 (6.4%)	8 (7.5%)	0.80	3 (17.6%)	0 (0.0%)	0.02
TGC+CAR+AMG	10 (6.5%)	1 (2.1%)	9 (8.5%)	0.14	1 (5.9%)	0 (0.0%)	0.18
TGC+TZP+AMG	7 (4.6%)	0 (0.0%)	7 (6.6%)	0.07	0 (0.0%)	0 (0.0%)	NA
TGC+VAN	23 (15.0%)	5 (10.6%)	18 (17.0%)	0.31	2 (11.8%)	3 (10.0%)	0.85
TGC+TEC	27 (17.6%)	5 (10.6%)	22 (20.8%)	0.13	3 (17.6%)	2 (6.7%)	0.24

*AMG, Aminoglycoside; ARI, Acute Renal Injury; CAP, Community Acquired Pneumonia; CAR, Carbapenem; CHD, Chronic Hepatic disease; cIAI, complicated Intra-abdominal Infection; CKD, Chronic Kidney Disease; COPD, Chronic Obstructive Pulmonary Disease; cSSTI, complicated Skin and Soft Tissue Infection; CST, Colistin; d, days; FDA, Food and Drug Administration; FN, Febrile Neutropenia; HAP, Hospital Acquired Pneumonia; HSCT, Hematopoietic Stem Cell Transplantation; NA, Not Applicable; SOFA, Sequential Organ Failure Assessment; TEC, Teicoplanin; TGC, Tigecycline; TZP, Piperacillin/Tazobactam; UTI, Urinary Tract Infection; VAN, Vancomycin; VAP, Ventilator Associated Pneumonia; 3GC, Third Generation Cephalosporin; 4GC, Fourth Generation Cephalosporin*.

N.B. Calculation of clinical and microbiological success and failure rates for all categories is done as such:

*1-Clinical success rate (%) = Number of patients with clinical success/(Number of patients with clinical success + Number of patients with clinical failure) × 100*.

*2-Microbiological success rate (%) = Number of patients with microbiological success / (Number of patients with microbiological success + Number of patients with microbiological failure) × 100*.

Critically ill patients had a significantly higher incidence of cardiovascular disease (77%, *P* = 0.003) compared to those outside critical care area. Also, the need for vasopressors and mechanical ventilation was equally higher in this category (38%, *P* = 0.01 and 36%, *P* = 0.01, respectively) compared with the other group. In the critically ill group, the incidence of cardiovascular disease, acute renal injury, as well as the need for vasopressors was significantly higher in the sicker category of these patients, i.e., those with SOFA ≥7 (87%, *P* = 0.03; 37%, *P* = 0.02 and 53%, *P* = 0.05, respectively) in comparison to those with a SOFA score <7.

### Tigecycline consumption and duration of therapy

During 2012 and 2013, respectively, tigecycline consumption reached 2.6 DDD/100BD and 4 DDD/100 BD in our facility. The majority of the patients received tigecycline for a duration of between 72 h and 10 days (64%), with a median duration of 8 days (Table 2). According to each indication, the median duration of therapy varied between 6.5 and 9.5 days. The range varied from 3 to 30 days. The longest duration of tigecycline therapy was seen in cases with febrile neutropenia (median 7 days; range 3–30 days; Table 2).

**Table 2:** **Clinical outcome, microbiological outcome and mortality in patients treated with tigecycline according to FDA approved and non-approved indications**.

**Indication**	**Duration of treatment (median; range) (Days)**	***T***	**Clinical Outcome**		**Microbiological Outcome**		**Mortality**		
			**Success**	**Outcome NR**	**Success**	**Outcome NR**	**7 days post-treatment mortality**	**In-hospital mortality**	**Total Mortality**
***FDA approved Indications***		29	16/25 (64%)	4	6/12 (50%)	17	3/29 (10.3%)	2/29 (6.9%)	5/29 (17.2%)
cSSTI	8 d; 3–18 d	18	7/15 (46.7%)	3	4/9 (44.4%)	9	3/18 (16.7%)	1/18 (5.6%)	4/18 (22.2%)
cIAI	6.5 d; 4–16 d	4	3/4 (75%)	0	1/2 (50%)	2	0	0	0
CAP	7 d; 4–12 d	8	6/7 (85.7%)	1	1/2 (50%)	6	1/8 (12.5%)	0	1/8 (12.5%)
*Off-label Indications*		124	43/111 (38.7%)	13	20/48 (41.7%)	76	48/124 (38.7%)	16/124 (12.9%)	64/124 (51.6%)
HAP	8 d; 3–26 d	30	8/28 (28.6%)	2	3/12 (25%)	18	14/30 (46.7%)	8/30 (26.7%)	22/30 (73.3%)
VAP	8 d; 3–19 d	39	9/35 (25.7%)	4	7/12 (58.3%)	19	16/39 (41%)	7/39 (18%)	23/39 (59%)
Bacteremia	8 d; 3–26 d	23	7/21 (33.3%)	2	4/18 (22.2%)	5	6/23 (26.1%)	0	6/23 (26.1%)
Sepsis	9 d; 3–26 d	14	1/14 (7.1%)	0	0	13	13/14 (92.9%)	0	13/14 (92.9%)
Diabetic Ulcer	9 d; 3–19 d	8	5/8 (62.5%)	0	3/5 (60%)	3	1/8 (12.5%)	0	1/8 (12.5%)
UTI	9.5 d; 3–24 d	6	4/5 (80%)	1	3/4 (75%)	2	1/6 (16.7%)	0	1/6 (16.7%)
FN	7 d; 3–30 d	24	16/19 (84.2%)	5	4/4 (100%)	20	3/24 (12.5%)	0	3/24 (12.5%)

*CAP, Community Acquired Pneumonia; cIAI, complicated Intra-abdominal Infection; cSSTI, complicated Skin and Soft Tissue Infection; d, days; FDA, Food and Drug Administration; FN, Febrile Neutropenia; HAP, Hospital Acquired Pneumonia; NR, Not Reported; T, Total; UTI, Urinary Tract Infection; VAP, Ventilator Associated Pneumonia*.

*Microbiological Success Rate (per each indication) = Number of patients with microbiological success/(T-Outcome NA) × 100*.

*Clinical Success Rate (per each indication) = Number of patients with clinical success/(T-Outcome NA) × 100*.

In the Mortality section, the denominator is always the total per each indication. The numerator in the “total mortality” is the sum of that in the “7 days post-treatment mortality” and that of the “in-hospital mortality.”

### Site of care

During the study period, the number of patient days was 89,800 in the hospital and 2777 in the critical care unit. Tigecycline was prescribed much more outside the critical care unit than inside it (106 patients vs. 47 patients, respectively; Table 1). However, on adjusting these values to patient days, the number of tigecycline cycles was 16/1,000 patient days inside the critical care unit and 1.2/1,000 patient days outside it. Inside the critical care area, tigecycline was mostly prescribed to patients with a SOFA score ≥7 (30/47 patients, 64% of the critically ill category). These patients have been at a higher risk of contracting multidrug-resistant *A. baumannii* infection compared with patients. According to our infection control data, it has been endemic in the critical care area of our facility.

### Indication for tigecycline

Tigecycline was prescribed more in off-label indications (124/153 patients, 81%) than in FDA-approved indications (29/153 patients, 19%; Table 1).

#### FDA-approved indications

Overall, cSSTIs were the most common (18/29, 62.1%) infections in this category followed by CAP (8/29, 27.6%). Inside the critical care area, CAP was the most common FDA-approved indication (4/6 patients, 66.7%) and all patients who were treated with tigecycline for an FDA-approved indication had a SOFA score <7 (6/17, 35.3%). Outside the critical care area, tigecycline was prescribed mostly in cSSTIs (23/29, 79.3%) compared with cIAI (4/29, 13.8%) and CAP (4/29, 13.8%).

#### Off-label indications

In general, VAP was the most common indication (39/124 patients, 31.5%), followed by HAP (30/124, 24.2%). Irrespective of the SOFA score, half of the critically ill patients treated with tigecycline had VAP (24/47 patients, 51.1%). Outside critical care, off-label indications were also a majority (83/106 patients, 78%). Fever with neutropenia represented the leading indication (23/83 patients, 27.7%; Table 1).

### Strategy of treatment

Tigecycline was used as targeted therapy in 56% of the cases (86/153 patients) and as empiric therapy in 44% (67/153 patients; Table 1). Empiric use was significantly higher outside critical care (49%, *P* = 0.049), unlike targeted therapy, which was significantly higher inside critical care area (68%, *P* = 0.049).

Tigecycline was combined with other antibiotics in 89% (136/153) of the cases without statistically significant difference between groups. Overall, tigecycline was most frequently combined with carbapenems (51/153, 46%) followed by colistin (47/153 patients, 30.7%) and piperacillin/tazobactam (38/153 patients, 24.8%).

### Clinical outcome

Because of the retrospective nature of the study, clinical success rates were calculated in patients with a known clinical outcome. Clinical success was achieved in 43.4% of the whole patient population (59/136 patients; Table 3). It was significantly higher in patients treated outside critical care compared to those inside ICU at the univariate level (49.5 vs. 31%, *P* = 0.04). Yet, the adjusted multivariate model failed to confirm this finding, where there was an insignificant difference between the two groups upon controlling for the following potentially confounding variables (OR = 1.07 [0.45–2.57], *P* = 0.87; Table 3).

**Table 3:** **Univariate and multivariate analysis for clinical outcome, microbiological outcome and mortality in patients treated with tigecycline inside vs. outside the critical care area**.

**Variables**	**Total (*N* = 153)**	**Univariate model**			Adjusted multivariate model^*^	
		**Critically ill patients (*n* = 47)**	**Patients outside critical care area (*n* = 106)**	***P***	**Odds ratio (95 % confidence interval)**	***P***
Clinical						
Outcome						
*Success*	59 (43.4%)	14 (31.1%)	45 (49.5%)	0.04	1.07 (0.45–2.57)	0.87
*Failure*	77 (56.6%)	31 (68.9%)	46 (50.5%)			
Microbiological						
Outcome						
*Success*	26 (43.3%)	8 (36.4%)	18 (47.4%)	0.41	0.96 (0.29–3.26)	0.95
*Failure*	34 (56.7%)	14 (63.6%)	20 (52.6%)			
Total mortality	69 (45.1%)	25 (53.2%)	44 (41.5%)	0.18	0.82 (0.36–1.89)	0.65
7 days post-treatment mortality	51 (33.3%)	20 (42.6%)	31 (29.2%)	0.11	1.15 (0.52–2.54)	0.73
In-hospital mortality	18 (11.8%)	5 (10.6%)	13 (12.3%)	0.77	0.85 (0.28–2.54)	0.77

Potentially confounding variables controlled for in the adjusted model

* *were: cardiovascular disease, vasopressors given before or ≤ 24 h of tigecycline therapy, mechanical ventilation, neutropenia (<500 mm^3^), ventilator associated pneumonia, treatment strategy; combination therapy using tigecycline plus aminoglycoside*.

N.B. Calculation of clinical and microbiological success and failure rates for all categories is done as such:

*1-Clinical success rate (%) = Number of patients with clinical success/(Number of patients with clinical success + Number of patients with clinical failure) × 100*.

*2-Microbiological success rate (%) = Number of patients with microbiological success/(Number of patients with microbiological success + Number of patients with microbiological failure) × 100*.

In the critically ill category, clinical success was significantly higher in patients with a SOFA score <7 compared with those with a SOFA score ≥7 using both univariate (62.5 vs. 13.8%, *P* = 0.003) and multivariate (OR = 12.51 [4.29–36.51], *P* < 0.0001) models (Table 4).

**Table 4:** **Univariate and multivariate analysis for clinical outcome, microbiological outcome and mortality in patients with SOFA score <7 and ≥7 receiving tigecycline therapy inside the critical care area**.

**Variables**	**Critically ill patients (*n* = 47)**	**Univariate model**			Adjusted multivariate model^*^	
		**SOFA score < 7 (*n* = 17)**	**SOFA ≥7 (*n* = 30)**	***P***	**Odds ratio (95 % confidence interval)**	***P***
Clinical						
Outcome						
*Success*	14 (31.1%)	10 (62.5%)	4 (13.8%)	0.001	12.51 (4.29–36.51)	<0.0001
*Failure*	31 (68.9%)	6 (37.5%)	25 (86.2%)			
Microbiological						
Outcome						
*Success*	8 (36.4%)	3 (33.3%)	5 (38.5%)	0.81	1.29 (0.37–4.54)	0.69
*Failure*	14 (63.6%)	6 (66.7%)	8 (61.5%)			
Total mortality	25 (53.2%)	2 (11.8%)	23 (76.7%)	<0.0001	5.17 (2.43–11.01)	<0.0001
7 days post-treatment mortality	20 (42.6%)	0 (0.0%)	20 (66.7%)	<0.0001	6.41 (3.04–13.52)	<0.0001
In-hospital mortality	5 (10.6%)	2 (11.8%)	3 (10.0%)	0.85	1.32 (0.63–2.77)	0.46

Potentially confounding variables controlled for in the adjusted model

* *were: age, cardiovascular disease, FDA-approved indications, off-label indications, community acquired pneumonia, and combination therapy using tigecycline plus aminoglycoside plus colistin*.

N.B. Calculation of clinical and microbiological success and failure rates for all categories is done as such:

*1-Clinical success rate (%) = Number of patients with clinical success/(Number of patients with clinical success + Number of patients with clinical failure) × 100*.

*2-Microbiological success rate (%) = Number of patients with microbiological success/(Number of patients with microbiological success + Number of patients with microbiological failure) × 100*.

With respect to the indication, clinical success was significantly higher in the FDA-approved group compared with the off-label group through univariate analysis (64 vs. 38.7%, *P* = 0.02). However, this finding was not confirmed by multivariate analysis (Table 5).

**Table 5:** **Univariate and multivariate analysis for clinical outcome, microbiological outcome, and mortality in FDA-approved vs. off-label indications**.

**Variables**	**Univariate model**			Adjusted multivariate model^*^	
	**FDA-approved indications (*n* = 29)**	**Off-label indications (*n* = 124)**	***P***	**Odds ratio (95 % confidence interval)**	***P***
Clinical					
Outcome					
*Success*	16 (64%)	43 (38.7%)	0.02	1.65 (0.60–4.49)	0.33
*Failure*	9 (36%)	68 (61.3%)			
Microbiological Outcome					
*Success*	6 (50%)	20 (16.1%)	0.58	1.07 (0.38–2.97)	0.90
*Failure*	6 (50%)	104 (83.9%)			
Total mortality	5 (17.2%)	64 (51.6%)	0.001	4.00 (1.30–12.31)	0.01
7 days post-treatment mortality	3 (10.3%)	48 (38.7%)	0.004	3.50 (0.97–12.61)	0.05
In-hospital mortality	2 (6.9%)	16 (12.9%)	0.53	2.00 (0.43–9.23)	0.37

Potentially confounding variables controlled for in the adjusted model

* *were: cardiovascular disease, vasopressors given before or ≤ 24 h of tigecycline therapy, hemodynamic failure, mechanical ventilation, treatment strategy, monotherapy, and combination therapy*.

CAP showed the highest success rate within the FDA-approved category (85.7%), whereas febrile neutropenia showed the best clinical outcome in the off-label category (84.2%). Septic patients receiving tigecycline showed the lowest clinical success rate (1/14 patients, 7.1%; Table 2).

### Microbiological outcome

Microbiological success rate was determined in cases in which follow-up cultures were available from patients' charts. Microbiological eradication of infection was achieved in 43.3% of patients who received tigecycline therapy (Table 3).

Yet, the univariate and multivariate models failed to show a significant difference in this outcome between patients inside vs. outside critical care area, those with SOFA score <7 vs. ≥7, and in FDA-approved vs. off-label indications (Tables 3, 4, 5).

### Mortality

Total mortality rate reached 45% in this study including 33% 7 day post-treatment mortality and 12% in hospital mortality. Findings of univariate and multivariate analysis show that there was not any statistically significant difference in these three types of mortality between patients inside and outside critical care (Table 3).

However, upon comparing patients with SOFA score ≥7 and those with SOFA score <7, only total mortality and 7 day post-treatment mortality were significantly higher in patients in the former group at the univariate (*P* < 0.0001) and multivariate levels (OR = 5.17 [2.43–11.01], *P* < 0.0001; OR = 6.41 [3.04–13.52], *P* < 0.0001, respectively; Table 4).

With respect to the indication, total mortality, and 7 days post-treatment mortality rates were significantly higher in the off-label indication group compared to the FDA-approved group, using univariate analysis (51.6 vs. 17.2%, *P* = 0.001; 38.7 vs. 10.3%, *P* = 0.004, respectively). However, the adjusted multivariate model confirmed this result for total mortality only (OR = 4.00 [1.30–12.31], *P* = 0.01; Table 5).

### Microbiological findings and isolate susceptibility to tigecycline

**Figure 1:**
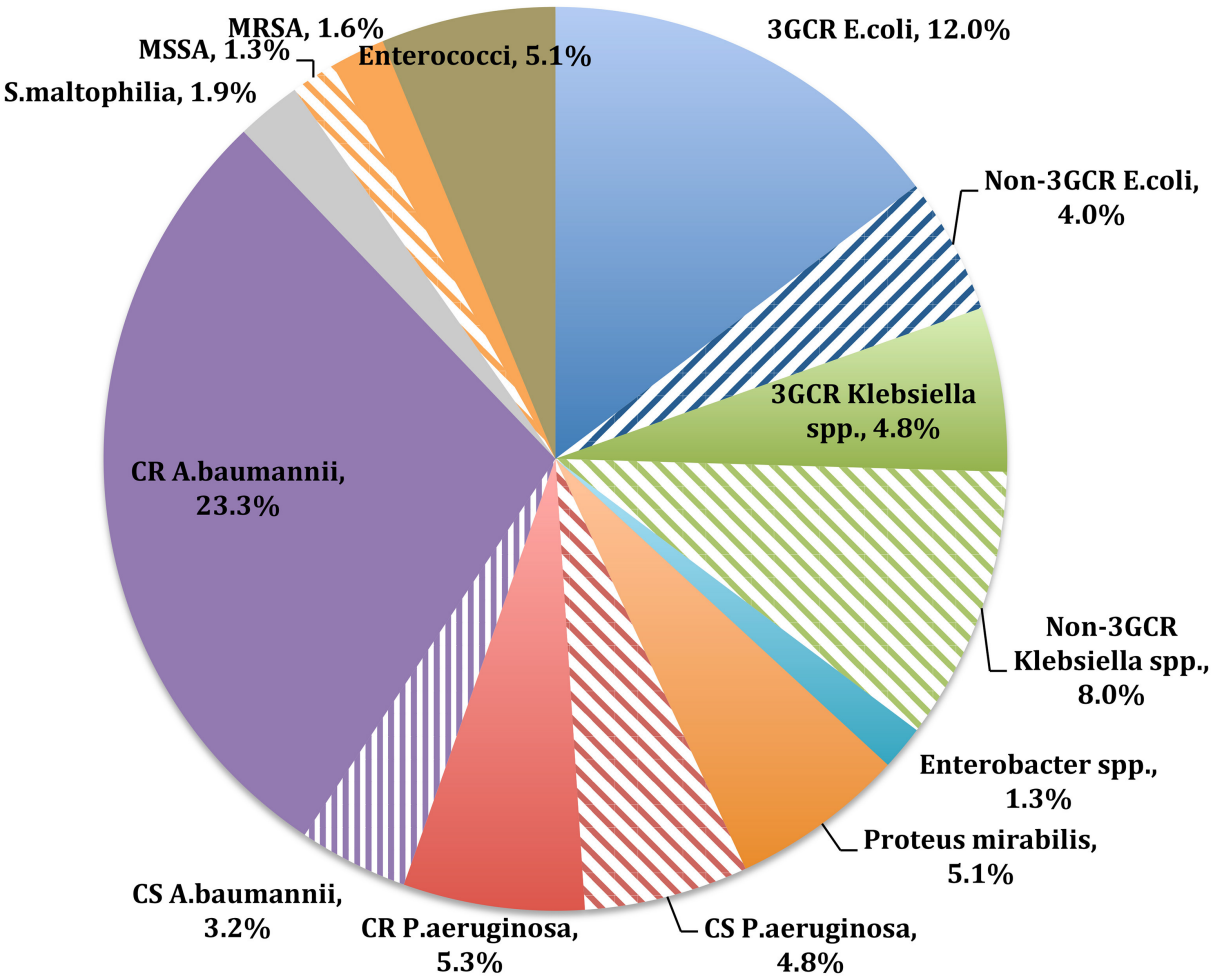
**Distribution of gram-positive and gram-negative bacteria isolated from clinical specimens during the study period**. MRSA, methicillin resistant *Staphylococcus aureus;* MSSA, methicillin sensitive *Staphylococcus aureus*; CR, carbapenem resistant; CS, carbapenem sensitive; 3GCR, third generation cephalosporin.

Distribution of gram-positive and gram-negative bacteria isolated from clinical specimens during the study period is shown in Figure 1. Gram-negative bacteria represented the majority of the clinical isolates (304/374 isolates, 81%; Supplementary File 2). Susceptibility to tigecycline reached 89% (194/217 isolates) in the gram-negative species and 91% (20/22 isolates) in the gram-positive species, as determined by the disc-diffusion method. *A. baumannii* (105/374 isolates, 28%) was predominant among the gram-negative species, of which 85% (87/102 isolates) were resistant to carbapenems. Yet, 82% (71/87 isolates) of the carbapenem-resistant *Acinetobacter* species were susceptible to tigecycline (XDR-AB). *Pseudomonas* species susceptibility to tigecycline was not tested (Table 4).

*E. coli* and *Klebsiella* species represented 16% (60/374 isolates) and 13% (48/374 isolates) of the total isolates, respectively. No carbapenem resistance was detected in either species. Overall, 98% (54/55 isolates) of the *E. coli* isolates were susceptible to tigecycline. Further, 77% of the *E. coli* isolates (46/60 isolates) were third-generation cephalosporin (3GC) resistant, of which 98% (43/44 isolates) were susceptible to tigecycline. With respect to *Klebsiella* species, 87% (40/46 isolates) were susceptible to tigecycline. Approximately 39% (18/48 isolates) were resistant to 3GCs, of which 78% (14/18 isolates) were tigecycline sensitive. *P. aeruginosa* was present in 12% (45/374 isolates) of the cultured bacteria, where 9% (4/45 isolates) were ceftazidime resistant and 44% (20/45 isolates) were carbapenem resistant (Table 4).

Among gram-positive bacteria, *Enterococci* represented 5% (19/374) of the total isolates, where none was vancomycin resistant. Tigecycline susceptibility was 83% (10/12) in this group. *Staphylococcus aureus* represented 3% (11/374) of the total isolates, where 55% (6/11) were methicillin resistant. All *S. aureus* strains were susceptible to tigecycline.

## Discussion

This study describes a single-center experience of tigecycline use in hospitalized patients in the absence of specific institutional guidelines for antibiotic use. It has been used in both FDA-approved indications such as cSSTI, cIAI, and CAP, and in off-label indications such as nosocomial pneumonia, bacteremia, febrile neutropenia, and UTI. Clinical and microbiological success rates were higher for the approved indications compared with the non-approved ones: 64 and 50% vs. 39 and 42%, respectively. In addition, total mortality was found to be significantly higher in the off-label indications compared with FDA-approved indications (OR= 4.00 [1.30–12.31], *P* = 0.01).

Upon comparing our results to pivotal studies from the literature, we found higher success rates in patients treated with tigecycline for FDA-approved indications as reported by Babinchack et al. for cIAI (80%; Babinchak et al., 2005), Ellis-Grosse et al. for cSSTI (80%; Ellis-Grosse et al., 2005), and Tanaseanu et al. for CAP (81%; Tanaseanu et al., 2008). This difference in patient outcome could be due to two factors. First, one half of our patients were critically ill and two thirds of them had a SOFA score >7. Second, the recovered bacterial species in our study were mostly MDR and XDR gram-negative species. Comparatively, in the aforementioned studies, the organisms were mostly gram-positive species in cSSTIs and antibiotic-susceptible gram-negative species in cIAIs.

In the study by Babinchak et al. (2005), the mortality rate in the tigecycline arm was higher than that of the comparator arm, imipenem-cilastatin. This study stated that the majority of patients who died had serious underlying preexisting conditions, tended to be over 65 years of age, and had relatively high severity-of-illness scores (Babinchak et al., 2005). Subsequently, in September 2010, the FDA issued a black box warning for an increased risk of death with the use of tigecycline, based on a pooled analysis of 13 phase III and phase IV clinical trials for both approved and unapproved indications (US FDA, 2010). In a recent study reviewing the *in vitro* susceptibility of tigecycline and comparators against gram-positive and gram-negative isolates collected from the Middle East and Africa between 2004 and 2011, the authors found that minimal inhibitory concentration (MIC 90) of ESBL-producing *E. coli* and *Klebsiella* species is higher than that in non-ESBL-producing species (Kanj et al., 2014). Given the fact that tigecycline highly concentrates in the biliary tree and moderately in the colon (De Rosa et al., 2015), we recommend its use only in non-critically ill patients for intrabiliary infections using the standard FDA-approved dose and for extrabiliary intra-abdominal infections using a double dose (200 mg loading dose followed by a maintenance dose of 100 mg every 12 h), especially if ESBL-producing organisms are expected or documented.

As for cSSTIs, tigecycline showed non-inferiority to vancomycin-aztreonam combination in two phase III, double-blind studies in hospitalized adults (Ellis-Grosse et al., 2005). In these trials, gram-positive species were predominant, including 13% methicillin-resistant *S. aureus* and 8% vancomycin-sensitive *Enterococci*. The global MIC 90 of these organisms does not exceed 0.25 mg/L (Hoban et al., 2015); however, that of ESBL-producing *E. coli, Klebsiella* species, and *A. baumannii* is ~2 mg/L (Hoban et al., 2015). Based on the poor therapeutic outcome of tigecycline in our study and its low mean peak concentration in the skin (0.56 ± 0.25 mg/L) reported elsewhere (Stein et al., 2011), we suggest that a double dose of tigecycline be used in the treatment of cSSTIs when resistant gram-negative ecology similar to ours is suspected and when there is no other alternative.

The management of pneumonia using tigecycline is another thorny issue. Tigecycline was safe, effective, and non-inferior to levofloxacin (500 mg/d IV) in hospitalized patients with CAP, according to two phase III, double-blind trials (Tanaseanu et al., 2008). However, this dose of levofloxacin is no longer the standard comparator for non-inferiority. The Infectious Diseases Society of America (IDSA) guidelines on CAP state that the recommended dosage for levofloxacin is 750 mg daily (Mandell et al., 2007). In addition, the first investigation to assess the steady-state intrapulmonary concentrations of tigecycline in a population of critically ill patients with underlying pulmonary pathology showed that at 1 h after tigecycline infusion, its concentration was 0.01 mg/L in the epithelial lining fluid (ELF) and 1.43 mg/L in the alveolar cells (ACs; Burkhardt et al., 2009). It is well-known that the antibiotic concentration in ELF and ACs is an important indicator for treatment success in lower respiratory tract infections (Mouton et al., 2008). Despite its low tissue concentration, tigecycline has proven activity in cases of CAP in which *Streptococcus pneumoniae* and other atypical bacteria with low MIC (0.06 mg/L) are suspected (Hoban et al., 2015). However, when other organisms with higher MIC (2 mg/L) are suspected, such as non-lactose fermenting gram-negative rods in patients with pulmonary pathology or in cases of HAP, the use of tigecycline becomes questionable. Accordingly, we assume that this issue has played a role in lowering clinical and microbiological success rates in our cases of nosocomial pneumonia: 29 and 25%, respectively, for HAP; 26 and 58%, respectively, for VAP. Therefore, we recommend using tigecycline in CAP when MIC 90 of *S. pneumoniae* is ≤0.06 mg/L. If lactose non-fermenters are suspected, double-dose tigecycline is recommended, but only if there is no alternative.

The use of tigecycline for the treatment of bacteremia is not advisable because of its low serum concentration. Burkhardt et al. showed that tigecycline plasma concentrations reach 0.43 ± 0.19 mg/L at 1 h and 0.36 ± 0.2 mg/L at 4 h after infusion (Burkhardt et al., 2009). These values are lower than MIC 90 of most gram-negative bacteria and some of the gram-positive bacteria (Hoban et al., 2015).

The use of double-dose tigecycline was based on findings of a pharmacokinetic study of single and multiple doses administered to healthy subjects (Muralidharan et al., 2005). Doses ranging from 12.5 to 300 mg were infused over 1 to 4 h. Investigators concluded that there is a linear correlation between the tested dose range with the mean maximum concentration (C_max_) and area under the curve (AUC; Muralidharan et al., 2005). The highest tolerated dose was 200 mg per day (Muralidharan et al., 2005). However, a recent study showed that high doses of tigecycline are associated with decreased fibrinogen level along with prolonged prothrombin time, activated partial thromboplastin time, and thrombin time (Zhang et al., 2015).

The main driver behind using tigecycline in our institution is the absence of other suitable alternatives with a relatively safe toxicological profile and yet good activity against endemic strains of XDR-AB. *In vitro* activity of tigecycline was determined by disc diffusion method in our study. Conflicting evidence lies in the choice of the testing method, where disc diffusion, Etest, and VITEK® 2 may yield false results (Piewngam and Kiratisin, 2014). In addition, no interpretation data are available for tigecycline susceptibility against *A. baumannii* from CLSI (2016) or The European Committee on Antimicrobial Susceptibility Testing (2016), regardless of the testing method. Nevertheless, when tigecycline is used, CLSI (2016) and The European Committee on Antimicrobial Susceptibility Testing (2016) recommend measuring tigecycline MIC using the recognized standard of broth microdilution. Accordingly, we recommend using the broth microdilution method for determining *in vitro* activity of tigecycline against endemic strains of *Acinetobacter* in corresponding centers.

In our series, tigecycline was used in combination with other antibiotics based on the growing evidence in the literature that supports the use of combination therapy against MDR and XDR organisms. A retrospective cohort study of 236 adult patients with XDR-AB pneumonia showed that a colistin–tigecycline combination regimen was superior to colistin monotherapy (Khawcharoenporn et al., 2014). In an attempt to fight *A. baumannii*, measuring mutant prevention concentration (MPC) of an active antibiotic against it can show its propensity to develop resistance. MPC theory has been proposed as a new measure for propensity of an antimicrobial to develop resistance (Zhao and Drlica, 2001). One study assessed the tendency of *A. baumannii* to develop resistance through measuring MPC of tigecycline (Cui et al., 2010). Authors found that tigecycline MIC is much lower than MPC, which means that attainable pharmacodynamics with FDA-approved dose will select mutants for resistance (Cui et al., 2010). Subsequently, tigecycline monotherapy for XDR-AB is not advisable. Higher doses and combination therapy with other active drugs such as polymyxins or rifampicin might lead to promising results (Poulikakos et al., 2014). We urge that trials examining the optimal treatment of infections due to MDR and XDR *Acinetobacter* species be conducted. A potential study would be testing efficacy of double-dose tigecycline combined with colistin in cases of XDR *Acinetobacter*, in comparison with tigecycline monotherapy.

The limitation of this study lies in its retrospective non-comparative nature. Because tigecycline was used in combination with other antibiotics, the individual assessment of its activity and efficacy was quite difficult. Moreover, it was challenging to evaluate all patient outcomes, especially in the sick population suffering from multiple comorbidities, in the absence of a control group. Data from a single center study like ours might not be representative of the whole country. Nevertheless, it could reflect a national perspective on physicians' prescribing behaviors, especially that Lebanon is a small country where the same Infectious Diseases specialists work simultaneously in different hospitals and use the same prescription strategies. This study shows a real-life experience in developing countries where XDR organisms have emerged leaving no room for many antibiotics to act. Clinicians find themselves facing virulent pathogens with very limited treatment options, yet with a nephrotoxic and neurotoxic profile, namely colistin. This reality highlights the importance of fully engaging pharmacokinetic and pharmacodynamic principles to optimize antimicrobial treatment in the face of emerging resistance. Furthermore, each healthcare facility should properly employ exact microbiological techniques for testing *in vitro* susceptibility of its endemic strains.

## Conclusion

Based on our results, we found that tigecycline was mostly used in off-label indications and in patients with high severity of illness scores (SOFA score ≥7). Those patients showed the worse outcomes and highest mortality rates. A drug oriented comprehensive antibiotic stewardship program is needed to improve patients' outcomes, curb resistance, and decrease costs. It should include therapeutic pathways that assign tigecycline use to each indication according to the type of infecting organism: starting at the bench, reaching the bedside.

## Ethics statement

Makassed General Hospital Institutional Review Board committee approved this study. No informed consent was required due to the retrospective nature of the study. During the data collection phase, a special form was used where patient initials and case numbers were only included. At a later stage, a different number was assigned to each of our cases thus aiming at safeguarding patient privacy. Data entry and analysis as well as the drafting of the paper were done by all contributing authors, who are all part of the same facility.

## Author contributions

All authors have contributed equally to the analysis and interpretation of the study data as well as to the drafting of the article. RM made the primary contribution to the conception and design of the study and revising the draft critically for important intellectual content and IF, TH, MK, NE to the acquisition and collection of data. All authors gave the final approval of the article to be sent for publication and agreed to be accountable for all aspects of the paper in ensuring that questions related to the accuracy or integrity of any part of the work are appropriately investigated and resolved.

### Conflict of interest statement

The authors declare that the research was conducted in the absence of any commercial or financial relationships that could be construed as a potential conflict of interest.
