# Supplementary material for: Prescription Patterns for Tigecycline in Severely Ill Patients for Non-FDA Approved Indications in a Developing Country: A Compromised Outcome
Source: Front Microbiol. 2017 Mar 27;8:497. doi: 10.3389/fmicb.2017.00497 (PMC5366332; doi:10.3389/fmicb.2017.00497)
Supplement: Supplementary file 1 [file Table1.DOCX]

**Supplementary file S1**

**Comparison between results of broth microdilution and disc diffusion method for *Acinetobacter* susceptibility to tigecycline**

| *Acinetobacter baumannii* strains | Tigecycline Minimal Inhibitory Concentration Using Broth Microdilution Method (mg/L) | Tigecycline Susceptibility Using Disc Diffusion Method* |
| --- | --- | --- |
| No. 1 | 0.75 | S |
| No. 2 | 0.75 | S |
| No. 3 | 0.75 | S |
| No. 4 | 1 | S |
| No. 5 | 1 | S |
| No. 6 | 1.5 | S |
| No. 7 | 1.5 | S |
| No. 8 | 2 | S |
| No. 9 | 1 | S |
| No. 10 | 1 | S |
| No. 11 | 1.5 | S |

N.B.*Tigecycline clinical breakpoints using the disc-diffusion method against *A. baumanii* were those suggested by Jones et al. [18] (susceptible ≥16 mm, intermediate 13-15 mm, and resistant ≤12 mm).
