# Supplementary material for: Prescription Patterns for Tigecycline in Severely Ill Patients for Non-FDA Approved Indications in a Developing Country: A Compromised Outcome
Source: Front Microbiol. 2017 Mar 27;8:497. doi: 10.3389/fmicb.2017.00497 (PMC5366332; doi:10.3389/fmicb.2017.00497)
Supplement: Supplementary file 2 [file Table2.DOCX]

**Supplementary file S2**

**Baseline bacterial isolates distributed according to tigecycline susceptibility**

| **Bacteria** | **Tigecycline (S)** | **Tigecycline (I) or (R)** | **Tigecycline Susceptibility NA** | **Total (n=374,%)** |
| --- | --- | --- | --- | --- |
| **Gram-negative species** | **194 (89.4%^1^)** | **23** | **87** | **304(81.3%)** |
| ***Escherichia coli*** | 54 (98.2%) | 1 | 5 | 60 (16%) |
| 3GCR | 43 (97.7%) | 1 | 2 | 46 (12%) |
| Non-3GCR | 11 (100%) | 0 | 3 | 14 (4%) |
| Carbapenem sensitive | 53 (98.1%) | 1 | 2 | 56 (15%) |
| Carbapenem resistant | 0 | 0 | 0 | 0 |
| ***Klebsiella species*** | 40 (87%) | 6 | 2 | 48 (12.8%) |
| 3GCR | 14 (77.8%) | 4 | 0 | 18 (4.8%) |
| Non-3GCR | 26 (92.9%) | 2 | 2 | 30 (8%) |
| Carbapenem sensitive | 39 (88.6%) | 5 | 0 | 44(11.8%) |
| Carbapenem resistant | 0 | 0 | 0 | 0 |
| ***Enterobacter species*** | 4 (100%) | 0 | 1 | 5 (1.3%) |
| ***Proteus mirabilis*** | 0 | 0 | 19 | 19 (5.1%) |
| ***Pseudomonas aeruginosa*^2^** | NA | NA | 45 | 45 (12%) |
| Ceftazidime sensitive | NA | NA | 34 | 34 (9.1%) |
| Ceftazidime resistant | NA | NA | 4 | 4 (1.1%) |
| Carbapenem sensitive | NA | NA | 18 | 18 (4.8%) |
| Carbapenem resistant | NA | NA | 20 | 20 (5.3%) |
| ***Acinetobacter baumannii^3^*** | 87 (85.3%) | 15 | 3 | 105 (28.1%) |
| Carbapenem sensitive | 12 (100%) | 0 | 0 | 12 (3.2%) |
| Carbapenem resistant | 71 (82.6%) | 15 | 1 | 87 (23.3%) |
| ***Stenotrophomonas maltophilia*** | 0 | 1 | 6 | 7 (1.9%) |
| **Gram-positive species** | **20 (90.9%)** | **2** | **48** | **70(18.7%)** |
| ***Staphylococcus aureus*** | 8 (100%) | 0 | 3 | 11 (2.9%) |
| *MSSA* | 3 (100%) | 0 | 2 | 5 (1.3%) |
| *MRSA* | 5 (100%) | 0 | 1 | 6 (1.6%) |
| ***Enterococci*** | 10 (83.3%) | 2 | 7 | 19 (5.1%) |
| *VSE* | 9 (100%) | 0 | 0 | 9 (2.4%) |
| *VRE* | 0 | 0 | 0 | 0 |

***KEY:*** *3GCR= Third Generation Cephalosporin Resistant, I= Intermediate, MSSA= Methicillin Sensitive Staphylococcus aureus, MRSA= Methicillin Resistant Staphylococcus aureus, VSE= Vancomycin Sensitive Enterococci, NA= Not Available, R=Resistant, S= Susceptible.*

***N.B.*** *^1^ % of susceptibility to tigecycline= number of isolates susceptible to tigecycline/ (number of isolates susceptible to tigecycline +number of isolates intermediate or resistant to tigecycline) ×100.^2^Tigecycline susceptibility for Pseudomonas species is not tested.^3^Carbapebem resistant isolates were considered extensive drug resistant.*
